# Spectra analysis and live-neuron imaging of cyclic AMP binding domain fusing circularly permutated GFP: Data for violet light excitable cyclic AMP indicator

**DOI:** 10.1016/j.dib.2022.108441

**Published:** 2022-07-04

**Authors:** Seiko Kawata, Yuto Yamamoto, Naoto Saitoh

**Affiliations:** Department of Neurophysiology, Graduate School of Life and Medical Sciences, Doshisha University, Kyoto 610-0394, Japan

**Keywords:** cAMP, Fluorescence, Probe, cpGFP

## Abstract

Cyclic adenosine monophosphate (cyclic AMP) is a second messenger, which is involved in the regulation of various cellular processes, including neuronal firing rate, synaptic plasticity, axon formation and axon elongation in brain. Although the main molecules in the cAMP-mediated signaling pathway are well studied, the spatio-temporal dynamics of the cAMP remain to be elucidated. Live imaging is an informative tool to investigate the cell signaling dynamics. It allows continuous monitoring of a specific cell over a period of time. Thus, optical probes for cAMP are important tools for studying the dynamics of cAMP signaling. Multiple genetically encoded cAMP probes are available [1], [2], including Förster resonance energy transfer (FRET) based or circular permutated fluorescent protein (cpFP) based probes. cpFP-based probes have an advantage of easier handling than FRET-based probes caused by monomeric detection and smaller molecular size. However, there is no cAMP probe compatible with violet light excitation. Therefore, we fused violet light excitable cpGFP to cyclic nucleotide binding domain (CBD) in *E. coli* cAMP receptor protein. This construct successfully responded to cAMP concentration changes. We show here the spectra data and live-cell imaging data of the violet light excitable cAMP probe which can be used for multi-signal fluorescence imaging.

## Specifications Table

**Table:** 

Subject	Biological sciences / Cell Biology
Specific subject area	Fluorescence imaging, Live-cell imaging, cAMP in cell detection
Type of data	Figure: PowerPoint file Spectrum data: Excel file Graph: Sigma Plot file Image: Zeiss file
How the data were acquired	Fluorescence spectrometer (FP-6500, JASCO) was used for spectra analysis. Samples were suspended in a buffer containing 50 mM Hepes/NaOH pH 7.0, 5 mM ascorbic acid, 4 mM glutathione and 0.1% bovine serum albumin. CBD-cpGFP expressing hippocampal neurons were imaged in HBSS. Confocal microscopy (LSM 710, Zeiss) was used for time-lapse imaging. Zen 2009 software was used for image capture and analysis.
Data format	Raw and Analyzed
Description of data collection	CBD-cpGFP protein was expressed in *E. coli* and purified with conventional His-Tag procedure. Purified CBD-cpGFP protein was analyzed using FP-6500 spectrometer. The fluorescence spectra showed an excitation peak at 404 nm and an emission peak at 512 nm, regardless of the presence or absence of cAMP. The CBD-cpGFP expressing HEK293T cells were imaged under confocal microscopy (Zeiss, LSM 710). 10 µM isoproterenol in HBSS was bath-applied using peristaltic pump at room-temperature.
Data source location	• *Institution:* Doshisha University • *City/Town/Region:* Kyotanabe/ Kyoto • *Country:* Japan
Data accessibility	With the article Repository name: “DIB-D-21-01837″, Mendeley Data Data identification number: 17632/h5wgkrfsk4.1 Direct URL to data: https://data.mendeley.com/datasets/h5wgkrfsk4/1

**Value of the Data**

• cAMP acts as second messengers and is widely present in many cell types such as neurons. cAMP fluorescent indicator allows continuous monitoring of a specific cell over a period of time.
• Almost all other signaling fluorescent indicators including FRET-based probes use blue or green light for their excitation. Thus, this cAMP indicator construct can easily combine with other indicators.
• Further mutations or modifications in this construct will expand the utility of violet excitable cAMP probe.

## 1 Data Description

**Fig. 1:**
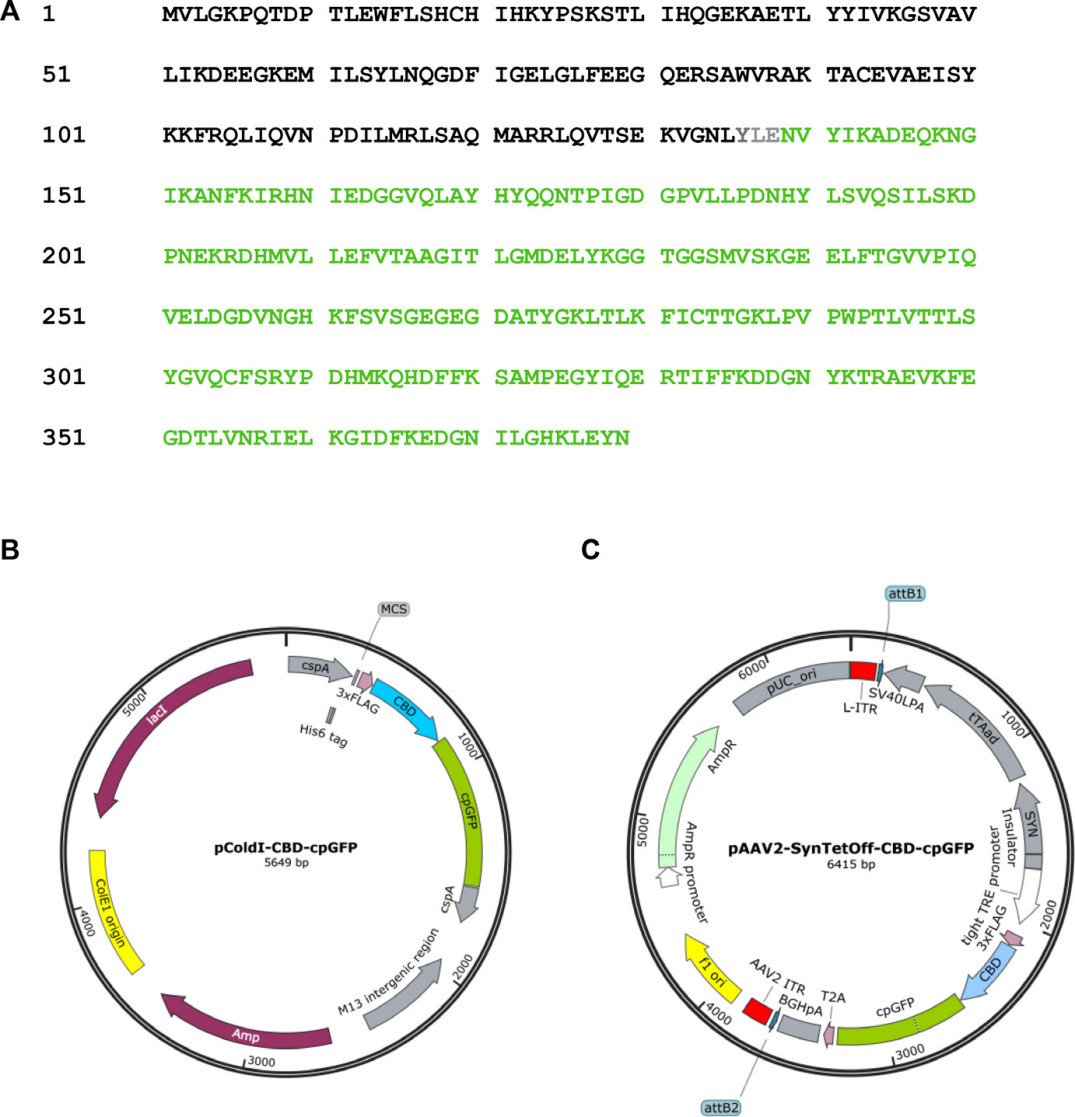
(A) Amino acid sequence of violet light excitable cAMP indicator. CBD sequence (in black) is based on *E. coli* CRP. Circularly permutated GFP sequence is highlighted in green. (B) Vector map of pColdI-CBD-cpGFP. (C) Vector map of pAAV2-SynTetOff-CBD-cpGFP.

**Fig. 2:**
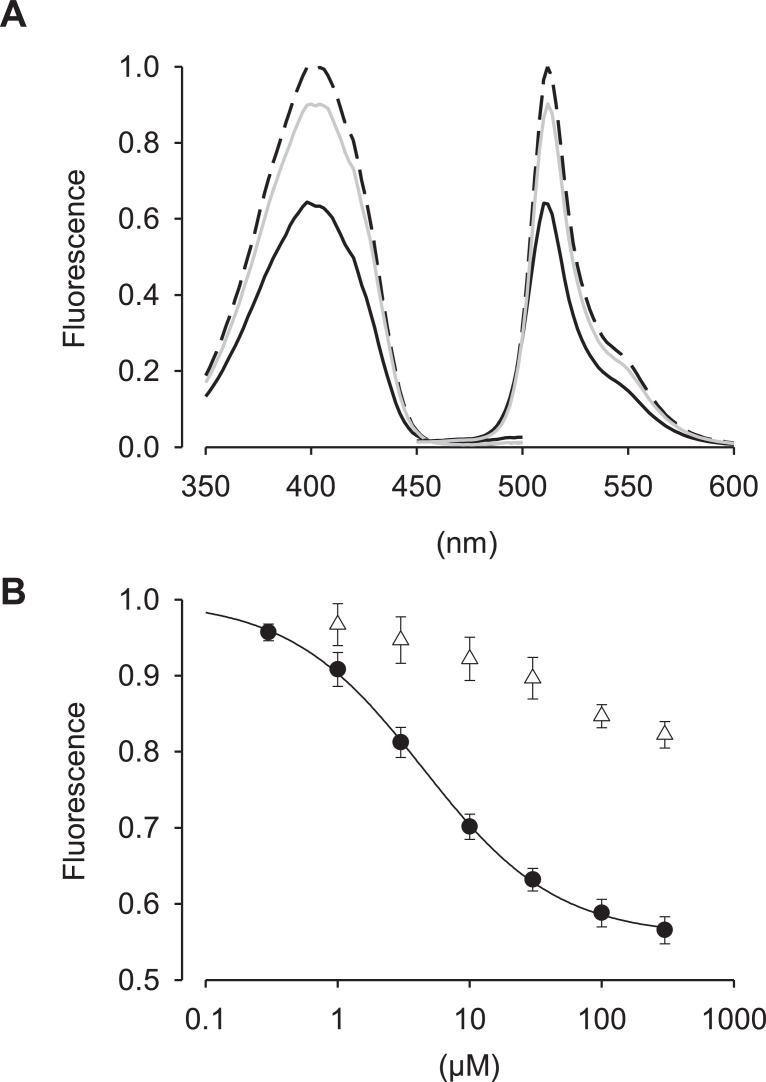
(A) Excitation (left) and emission (right) spectra in the absence (dashed line) or presence of 30 µM cAMP (solid line) or 30 µM cGMP (gray solid line). Each mean spectrum was normalized to the peak of fluorescent intensity in the absence of cAMP (*n* = 4 independent experiments). (B) Dose–response plots for cAMP (closed circle) and cGMP (open triangle). Data represent mean ± SD (*n* = 4 independent experiments). Fitting curves for cAMP (solid line) was calculated with Hill equation. The dissociation constant was 4.4 ± 0.6 µM and the Hill coefficient was 0.86 ± 0.08.

**Fig. 3:**
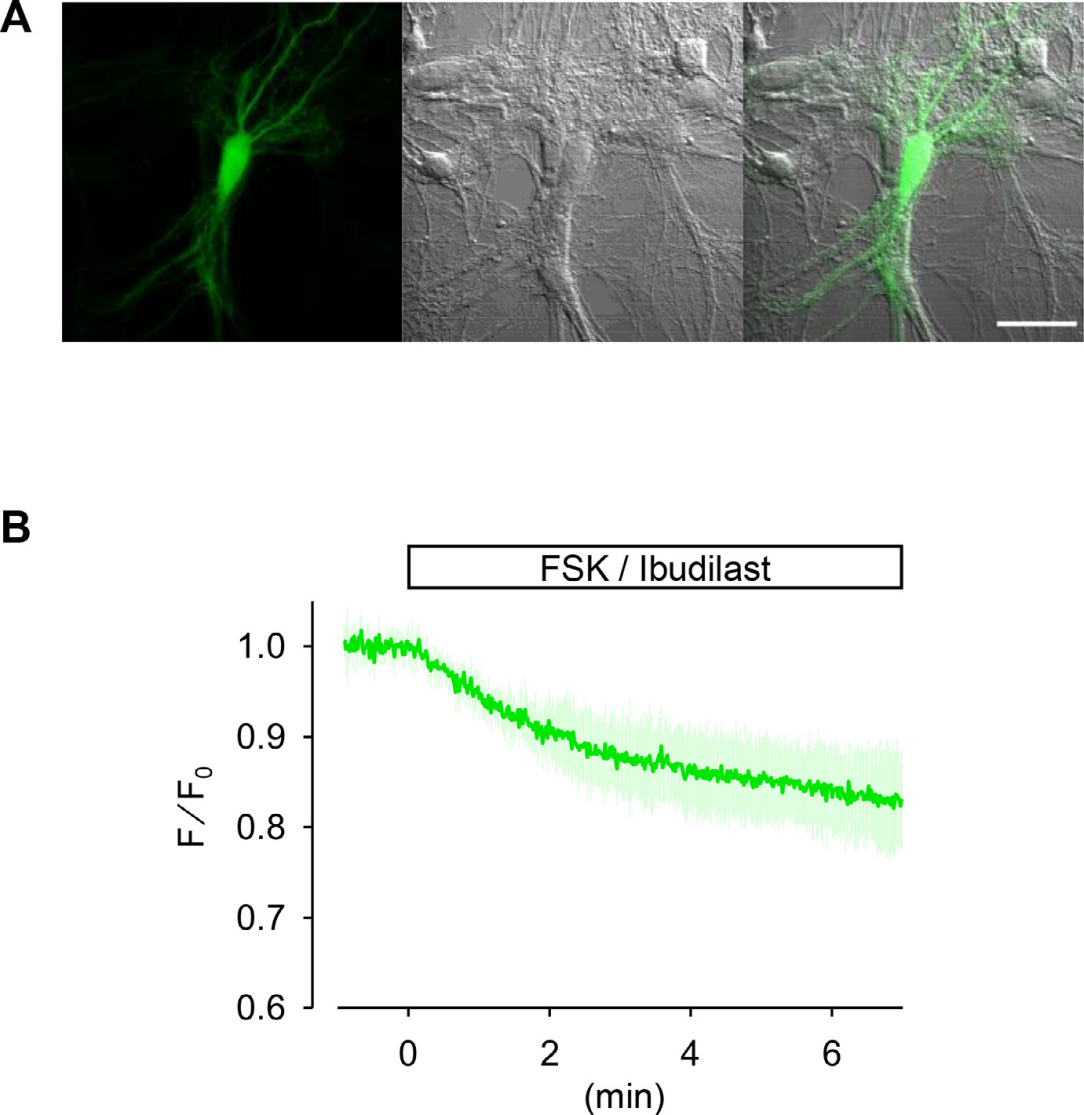
(A) The violet excitable cAMP probe was expressed in hippocampal neuron using rAAV-SynTetOff vector. Scale bar = 50 µm. (B) Hippocampal neurons were stimulated by 10 µM forskolin / 100 µM ibudilast. Data represent mean ± SD (*n* = 8 cells from three independent experiments).

The *Escherichia coli* (*E. coli*) CRP is a transcriptional activator which regulates many transcription units in response to intracellular concentration of cAMP. CRP is organized in two distinct domains: (i) an N-terminal CBD (residues 1–136), which contains the cyclic nucleotide-binding module and (ii) a C-terminal DNA-binding domain (DBD; residues 139–209), which contains a helix-turn-helix motif for binding to DNA [3]. So, we designed the cAMP probe for which the CBD fuses to circularly permutated GFP (cpGFP) instead of the DBD (Fig. 1A). Then, the CBD-cpGFP was inserted into pColdI and pAAV2-SynTetOff [5] vectors for protein expression in *E. coli* and hippocampal neurons, respectively (Fig. 1B and C). The CBD-cpGFP protein was expressed in *E. coli* BL21, then purified CBD-cpGFP protein was analyzed spectra using fluorescence spectrometry in the presence or absence of cAMP or cGMP (Fig. 2A). The CBD-cpGFP fluorescence was decreased with cAMP elevation. Fitting curve (Fig. 2B) indicates the *K*_d_ = 4.4 µM (Hill coefficient = 0.86). The CBD-cpGFP was expressed in hippocampal neurons using rAAV vector (Fig. 3A). Hippocampal neurons (DIV18) were stimulated by 10 µM forskolin (agonist for adenylyl cyclase)/ 100 µM ibudilast (inhibitor for phosphodiesterase) under confocal microscopy. The CBD-cpGFP fluorescence was also decreased with cAMP elevation in hippocampal neurons (Fig. 3B).

The deposited data (doi: 10.17632/h5wgkrfsk4.1) contain as follows:

pColdI-CBD-cpGFP.dna: SnapGene DNA file of the CBD-cpGFP in pCold I *E.coli* expression vector.
pAAV2-SynTetOff-CBD-cpGFP.dna: SnapGene DNA file of the CBD-cpGFP in pAAV2-SynTetOff vector for neuronal expression.
CBD-cpGFP raw spectra data.xlsx: the excitation and emission spectra of the CBD-cpGFP protein in the absence or presence of cAMP.
CBD-cpGFP raw dose response.xlsx: the raw dose–response data of the CBD-cpGFP for cAMP or cGMP.
CBD-cpGFP raw time lapse.xlsx: the time-lapse raw data of the CBD-cpGFP expressing hippocampal neurons stimulated by the application of forskolin/ibudilast.
CBD-cpGFP analyzed spectrum and dose response.JNB: SigmaPlot graph file of the normalized mean excitation and emission spectra, and the normalized dose–response data for cAMP or cGMP.
CBD-cpGFP analyzed time lapse.JNB: SigmaPlot graph file of the normalized time-lapse data of the CBD-cpGFP expressing hippocampal neurons stimulated by the application of forskolin/ibudilast.
CBD-cpGFP in hippocampal neurons.lsm: Zeiss image file of CBD-cpGFP expressing hippocampal neuron.

## 2 Experimental Design, Materials and Methods

### 2.1 Plasmid Construction

Green fluorescent cAMP indicators were constructed as follows: cAMP-binding domains of E. coli (DH5a) CRP (a.a. 1–134, GenBank Accession Number KP670514) were PCR amplified (*Pfx50*, ThermoFisher). cpGFP of GEX-GECO [4] was also PCR amplified. These PCR products were inserted into subcloning vector pBluescript II KS(+) (Agilent), followed by sequence analysis. cpGFP was fused to the *Xho* I site attached to the C-terminal of CBD. CBD-cpGFP construct was inserted into pCold I (TaKaRa) and pAAV2-SynTetOff [5] for protein expression in *E. coli* and hippocampal neurons, respectively.

### 2.2 PCR Primers

CBD Forward: TAGGATCCATGGTGCTTGGCAAACCG.
CBD Reverse: TTACTCGAGGTACAGGTTGCCCAC.
cpGFP Forward: TACTCGAGAACGTCTATATCAAGGC.
cpGFP Reverse: TAAGTCGACAAGCTTACTAGTGTTGTACTCCAGCTTGTGCC.

2-step PCR (TP-600, Takara): 94 °C 15 s / 68 °C 40 s, 30 cycle.

### 2.3 Bacterial Protein Expression and Purification

*E. coli* BL21 (BioDynamics) transformed with histidine tag containing pCold I-CBD-cpGFP was grown at 37 °C until OD_600_≈0.4 in 50 mL LB medium supplemented with 100 µg/mL ampicillin, and protein expression was induced by adding 0.5 mM IPTG and incubating for an additional 20–24 h at 15 °C. Protein-expressing *E. coli* was collected and lysed with 3 mL BugBuster solution (Merck) for 30 min at 4 °C, followed by binding with 300 µL cobalt resin (ABT) with 30 mL buffer containing (in mM) 50 Na-phosphate, 300 NaCl, and 10 imidazole for 1 h at 4 °C. Protein-binding resin was washed thrice with the buffer (30 mL) using centrifuge. Protein was eluted with 3 mL elution buffer containing (in mM) 50 Na-phosphate, 300 NaCl, and 150 imidazole. Subsequently, protein was concentrated and its buffer replaced to phosphate buffered saline by 10,000 MW cut ultrafiltration (VIVASPIN 6, sartorius). The concentrated protein solutions were stored at 4 °C under dark conditions.

### 2.4 Optical Properties Analysis

Purified CBD-cpGFP protein was suspended in the buffer containing 50 mM HEPES pH 7.0, 5 mM ascorbic acid, 4 mM Glutathione and 0.1% bovine serum albumin. The fluorescence spectra were measured using a fluorescence spectrophotometer (FP-6500, JASCO).

### 2.5 Primary Hippocampal Culture

Hippocampi were dissected from ICR embryos at E17. Hippocampal neurons were dissociated by using Neuron Dissociation Solutions (Fujifilm) and plated on coverslips coated with poly-D-lysine at a density of 3 × 10^4^ cells/cm^2^ in Neurobasal plus medium supplemented with B27 (Thermo Fisher) and 2 mM L-alanyl-L-glutamine. Dissociated neurons were maintained at 37 °C in 5% CO_2_ for 3 weeks.

### 2.6 Recombinant AAV Preparation

pAAV-SynTetOff, pR2C1, and pHelper (Takara) were cotransfected into HEK293T cells (ATCC) by calcium phosphate transfection (Promega). The medium was replaced 6 h after transfection with Dulbecco's modified Eagle's medium (Fujifilm), containing 10% fetal bovine serum (JRH Biosciences). The medium containing virus particles were collected 72 h after medium replacement subsequently filtered by 0.45 µm PVDF membrane (Millex-HV, Millipore). The rAAV containing medium was added (20 µL / 3 × 10^4^ cells) to hippocampal neuronal cultures at 7 DIV.

### 2.7 Live-Cell Imaging

Time-lapse images were taken using a confocal microscopy (LSM710, Zeiss) with a 20×, 0.8 N.A. dry objective lens. 405 nm laser was used for excitation of cpGFP. Emission was collected between 489 nm and 538 nm. Time-lapse images were performed in Hank's balanced salt solution (Fujifilm) with/without 10 µM forskolin (Fujifilm) and 100 µM ibudilast (Fujifilm), and acquired every 1 s using Zen 2009 software (Zeiss).

## Ethics Statements

All experiments were performed in accordance with the DNA (#D19010, #D20064, #D21044 and #D22043) and animal care (#A21044 and #A22043) guidelines of Doshisha University.

## CRediT authorship contribution statement

**Seiko Kawata:** Investigation, Writing – original draft, Supervision. **Yuto Yamamoto:** Investigation. **Naoto Saitoh:** Writing – original draft, Project administration.

## Declaration of Competing Interest

The authors declare that they have no known competing financial interests or personal relationships that could have appeared to influence the work reported in this paper.

## Data Availability

DIB-D-21-01837 (Original data) (Mendeley Data).
